# Impact of hybrid assistive limb on gait ability and quality of life in patients with amyotrophic lateral sclerosis

**DOI:** 10.3389/fneur.2026.1823485

**Published:** 2026-10-14

**Authors:** Harumi Morioka, Takehisa Hirayama, Mari Shibukawa, Tatsuki Sugisawa, Junpei Nagasawa, Sayori Hanashiro, Junya Ebina, Ryuichi Okamoto, Makiko Ogawa, Ikuko Okuni, Yoshitaka Murakami, Osamu Kano

**Affiliations:** 1 Department of Neurology, Toho University Faculty of Medicine, Tokyo, Japan; 2 Department of Rehabilitation Medicine, Toho University Faculty of Medicine, Tokyo, Japan; 3 Department of Medical Statistics, Toho University Faculty of Medicine, Tokyo, Japan

**Keywords:** amyotrophic lateral sclerosis, cyborg robot, hybrid assistive limb, QoL, SF-36, gait ability

## Abstract

**Objective:**

Hybrid Assistive Limb (HAL) is a cyborg-type wearable robot that supports gait training based on the wearer’s intended motion. This study aimed to evaluate the effects of HAL treatment on gait ability and quality of life (QoL) in patients with amyotrophic lateral sclerosis (ALS).

**Methods:**

Patients with an unsteady gait with the ability to walk ≥10 m with assistance from a caregiver and/or walker were included in this study. Patients underwent nine HAL treatment sessions (20–40 min per session), with no more than one session per day, over a period of at least 4 weeks. Gait ability was evaluated using the distance of the 2-min walk test (2MWT), and the speed (m/s), step length (m/step), and cadence (step/s) of the 10-m walk test. Both tests were performed without HAL assistance. QoL was evaluated using the 36-Item Short Form Health Survey.

**Results:**

We evaluated 30 patients who completed one course of treatment. To evaluate the effectiveness of HAL, a paired t- test was used for the results of one course. With one course of treatment, the 2MWT improved from 92.27 m (40.46) at baseline to 104.16 m (41.73) (*p* < 0.001). Regarding QoL, the mental component summary score was 55.35 (8.27) before HAL treatment, indicating relatively preserved mental QoL. There were no significant changes in QoL before and after treatment.

**Conclusion:**

Although no curative treatment exists for ALS, HAL treatment may provide short-term improvement in gait ability in patients with ALS. However, no significant changes in QoL were observed after one course of treatment.

## 1 Introduction

Amyotrophic lateral sclerosis (ALS) is a progressive neurodegenerative disorder characterized by the degeneration of the upper and lower motor neurons. As the disease progresses, patients experience muscle weakness, leading to declines in gait function and activities of daily living.

Gait function is closely associated with quality of life (QoL) in patients with ALS, as it affects independence, mobility, and social participation (1, 2). Therefore, maintaining gait function is an important goal in clinical management. However, this relationship is complex, and changes in physical function do not necessarily translate into short-term changes in QoL. In particular, it has been reported that physical and mental components of QoL may diverge in ALS, with mental QoL remaining relatively stable despite deterioration in physical function (3). This suggests that changes in motor function do not always correspond to the changes in patient-reported outcomes.

Rehabilitation plays a central role in symptomatic management. While some studies have suggested benefits of rehabilitation (4), its effects on physical function and disease progression remain inconsistent (4–6), and only a few reports provide quantitative evaluations (7–9).

Robotic rehabilitation emerged approximately in the 1980s, and its development has progressed rapidly since 2000 (10). Various lower-limb exoskeleton devices have been introduced for gait rehabilitation. Hybrid Assistive Limb^®^ (HAL^®^, Cyberdyne Inc., Japan) is a wearable robotic device that assists voluntary movement based on bioelectrical signals (BES). This allows the user’s motor intention to be translated into assisted joint motion. The effects of HAL have been reported in stroke (11–20), spinal cord injury (SCI) (21–26), and cerebral palsy (27–32). For neuromuscular diseases, a randomized controlled crossover study (NCY-3001) was conducted (33). Results showed improvement in the 2-min walk test (2MWT), and in 2015, HAL lower limb type was approved as a medical treatment device in Japan for eight neuromuscular diseases: ALS, spinal muscular atrophy, spinal and bulbar muscular atrophy, Charcot–Marie–Tooth disease, muscular dystrophy, distal myopathy, sporadic inclusion body myositis, and congenital myopathy. Furthermore, in 2023, the approved indications for HAL lower limb type in Japan were expanded to include HTLV-1 associated myelopathy and hereditary spastic paraplegia. The medical use of HAL has also expanded beyond Japan. In the United States, the indication for HAL was expanded in 2020 to include progressive neuromuscular diseases, including ALS, and the device has also obtained regulatory certification in the European Union (33). However, these diseases are rare, and studies evaluating the efficacy of HAL treatment in ALS remain scarce (34–36), particularly in larger cohorts.

In our previous studies, maintenance of motor function was observed in patients with ALS following HAL treatment (35, 36). However, because ALS is a progressive disease, evaluating long-term maintenance of function is challenging in clinical practice. Therefore, short-term functional changes may provide preliminary insights, although their clinical significance remains limited.

Furthermore, it remains unclear whether such functional changes are associated with changes in patient-reported outcomes such as QoL. Therefore, this study aimed to evaluate short-term changes in gait function and QoL following a HAL-based rehabilitation program in a relatively large cohort of patients with ALS.

## 2 Materials and methods

This was a single-center, baseline-controlled observational study. This study was conducted at the Department of Neurology and Department of Rehabilitation Medicine, Toho University Faculty of Medicine (Tokyo, Japan). The current study was approved by the Ethics Committee of Toho University Omori Medical Center (No. M24067 21074 18122). Written informed consent was obtained from all the participants.

### 2.1 Participants

We enrolled patients diagnosed with ALS (according to the updated Awaji criteria of ALS) (37) by board-certified neurologists in our department between January 2019 and April 2023. The inclusion criteria were as follows (i) age ≥18 years, (ii) body type that can wear HAL (height, 150–190 cm; weight, 40–100 kg), (iii) no rapid change of gait in the past 3 months, and (iv) cannot walk for 10 m independently, but has the ability to walk for ≥10 m with assistance or walking aids. The exclusion criteria were as follows: (i) cognitive impairment (Mini-Mental State Examination [MMSE] score ≤20 points); (ii) difficulty in gait training due to severe systemic conditions; (iii) difficulty in gait due to cerebral and muscle disorders other than ALS; (iv) bruises and fractures that required hospitalization within 3 months prior to this study; (v) starting new gait training programs within the month prior to this study; (vi) starting treatment with steroids, riluzole, sodium valproate, and other drugs for controlling the progression of ALS; and (vii) inability to attach HAL sensor pads to the skin.

### 2.2 Treatment schedule

Treatment was performed by two physiotherapists and a doctor who was trained to use the HAL system. The treatment program using HAL consisted of one session per day (20–40 min per session, including setup and breaks), 2–3 sessions a week for at least 4 weeks but no more than 5, according to the patients’ conditions. One course of HAL treatment consisted of nine sessions. On days without HAL treatment, patients received conventional physical therapy without HAL (Figure 1).

**Figure 1:**
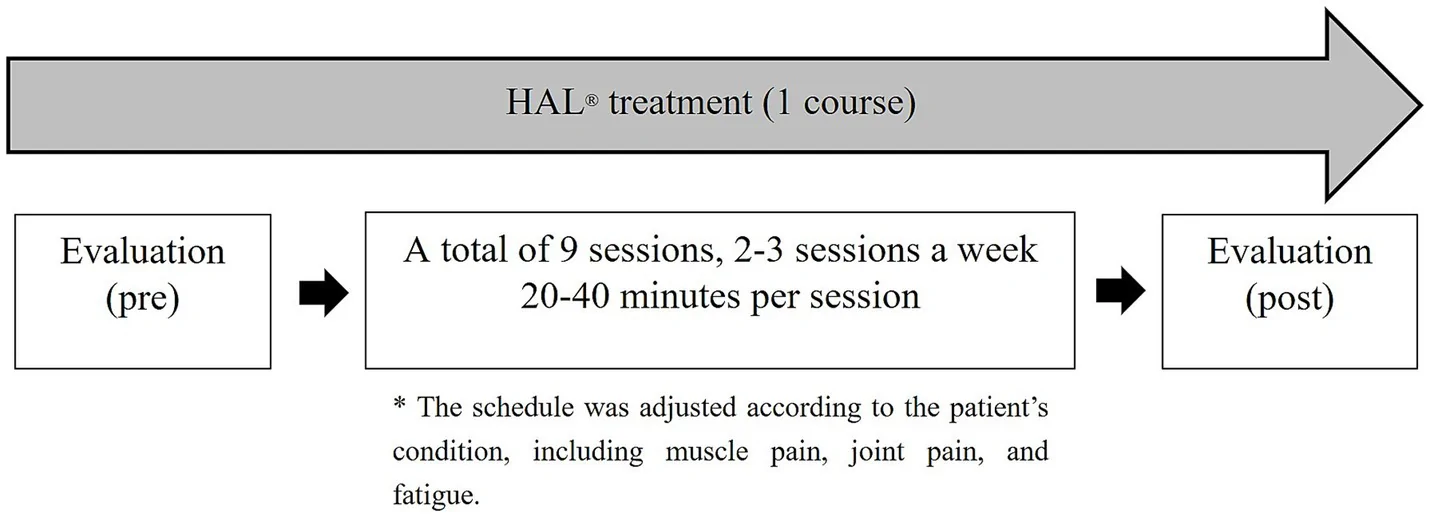
Study flow diagram. Patients underwent baseline assessment followed by a 4–5 week intervention including HAL treatment (9 sessions, 2–3 sessions per week, 20–40 min per session; including setup and rest periods) and conventional physical therapy. Outcome measures were assessed before and after the intervention.

The treatment program with HAL was implemented using a treadmill (manufacturer: NIHON KOHDEN CORPORATION). Training started with Cybernic Voluntary Control mode, which provides assistive torque based on BES, defined as weak electrical signals associated with the voluntary neuromuscular activity that can be detected on the skin surface (36). BES were recorded using surface electromyographic electrodes placed over the patients’ iliopsoas, quadriceps, hamstrings, and gluteal muscles. The settings were determined according to the severity of muscle contraction for each patient so that the patients could easily raise their lower extremities. During training, the physiotherapist checked the BES and adjusted the motor assist level for each patient. In addition, control parameters, including torque limit, balance, and gait support, were individually adjusted based on each patient’s motor function and clinical condition. Walking speed was also individually adjusted according to motor performance, fatigue, and safety considerations. For safety reasons, support from the handrail of the treadmill and a walking device with a harness were used, and two physiotherapists supported the patients and prevented falls. If patients experienced respiratory discomfort during training, or fatigue and muscle pain on the day following training, the treadmill speed was adjusted appropriately.

### 2.3 Outcome measures

Baseline measures included demographic data, pertaining to the following: site of onset, duration of ALS, the severity of ALS, % forced vital capacity (%FVC), riluzole prescription, percutaneous endoscopic gastrostomy (PEG), noninvasive ventilation (NIV), and MMSE score. Gait ability (2MWT and 10-m walk test [10MWT]) and QoL (36-Item Short Form Health Survey [SF-36]) were assessed prior to treatment. In the 2MWT, the distance which the patients could walk for 2 min was measured. In the 10MWT, the time was measured and the number of steps required to walk 10 m was counted to calculate walking speed (m/s) and step length (m/step). Cadence (steps/s) was calculated from the time and number of steps. In both the 2MWT and 10MWT, the patients walked on a flat floor at their self-selected maximum speed without wearing HAL. Assistance, such as handrails, walkers, and caregivers’ help, was allowed, if required. Gait ability was assessed by physical therapists trained to perform standardized assessment procedures. ALS severity was assessed using the ALS Functional Rating Scale-Revised (ALSFRS-R).These outcome measures were evaluated before and after one course of HAL treatment.

To evaluate the effectiveness of training with HAL on gait ability, the outcome measures, including the walking distance of the 2MWT, walking speed, step length, cadence of the 10MWT, and scores of SF-36, were compared before and after training using the paired t-test. Statistical significance was set at *p* < 0.05.

## 3 Results

Thirty patients participated in this study, and patient details are summarized in Table 1. This study comprised 17 male and 13 female patients, and the mean age (standard deviation) at baseline was 60.4 (12.8) years. The site of onset was bulbar in three patients, the upper extremities in seven, and the lower extremities in 20. The mean duration of ALS was 46.1 (37.3) months. The mean ALSFRS-R score was 39.4 (3.8), and the %FVC was 95.0% (14.8%). Of the 30 patients, two used NIV, and 25 were prescribed riluzole. None of the patients started using PEG or NIV during the observation period. All patients were assessed using the MMSE, and they had no cognitive dysfunction. There were no dropouts and treatment-related adverse events or accidents.

**Table 1:** Baseline characteristics of patients with ALS included in the study.

Characteristic	Value (*n* = 30)
Sex (Male/Female)	17/13
Age, years	60.4 (±12.8)
Onset, *n* (%)	
Bulbar	3 (10%)
Upper extremities	7 (23.3%)
Lower extremities	20 (66.7%)
Duration (months) (SD)	46.1 (37.3)
ALSFRS-R (SD)	39.4 (3.8)
%Forced vital capacity (SD)	95.0 (14.8)
Riluzole prescription	25 (83%)
PEG	0 (0%)
NIV	2 (7%)
MMSE	29.8 (0.8)

Data are presented as mean ± standard deviation or number (%), as appropriate. ALS, amyotrophic lateral sclerosis; ALSFRS-R, ALS Functional Rating Scale–Revised; PEG, percutaneous endoscopic gastrostomy; NIV, noninvasive ventilation; MMSE, Mini-Mental State Examination SD, standard deviation.

Data from the assessments at baseline and immediately after the completion of one course of treatment are presented in Table 2. Regarding the 2MWT, the mean distance significantly changed from 92.27 m (40.46) at baseline to 104.16 m (41.73) after one course of treatment (*p* < 0.001) (Figure 2). The effect size was small (Cohen’s d = 0.29). The mean walking time per HAL session was 11.78 min (4.47). The correlation between mean walking time per session and the change in 2MWT distance was weak (r = 0.082). Regarding the 10MWT, the average speed values were 0.93 m/s (0.40) at baseline and 0.95 m/s (0.38) after treatment (*p* = 0.407), with a negligible effect size (d = 0.05) (Figure 3). The average cadence values at baseline were 1.82 steps/s (0.48) and 1.83 steps/s (0.47) (*p* = 0.910) after treatment, and the effect size was negligible (*d* = 0.01) (Figure 3). The average step length changed after one course of treatment from 0.50 m/step (0.13) to 0.51 m/step (0.12) (*p* = 0.161), indicating a negligible effect size (*d* = 0.11) (Figure 3).

**Table 2:** Changes in walking parameters before and after one course of intervention.

Walk test	Parameter	Baseline	After one course	*p* value
2-min walk test	Distance (m)	92.27 (40.46)	104.16 (41.73)	<0.001
10-m walk test	Speed (m/s)	0.93 (0.40)	0.95 (0.38)	0.407
	Cadence (steps/s)	1.82 (0.48)	1.83 (0.47)	0.910
	Step length (m/step)	0.50 (0.13)	0.51 (0.12)	0.161

Data are presented as mean values (standard deviation). Baseline and post-intervention values were compared using paired *t*-tests. Statistical significance was set at *p* < 0.05.

**Figure 2:**
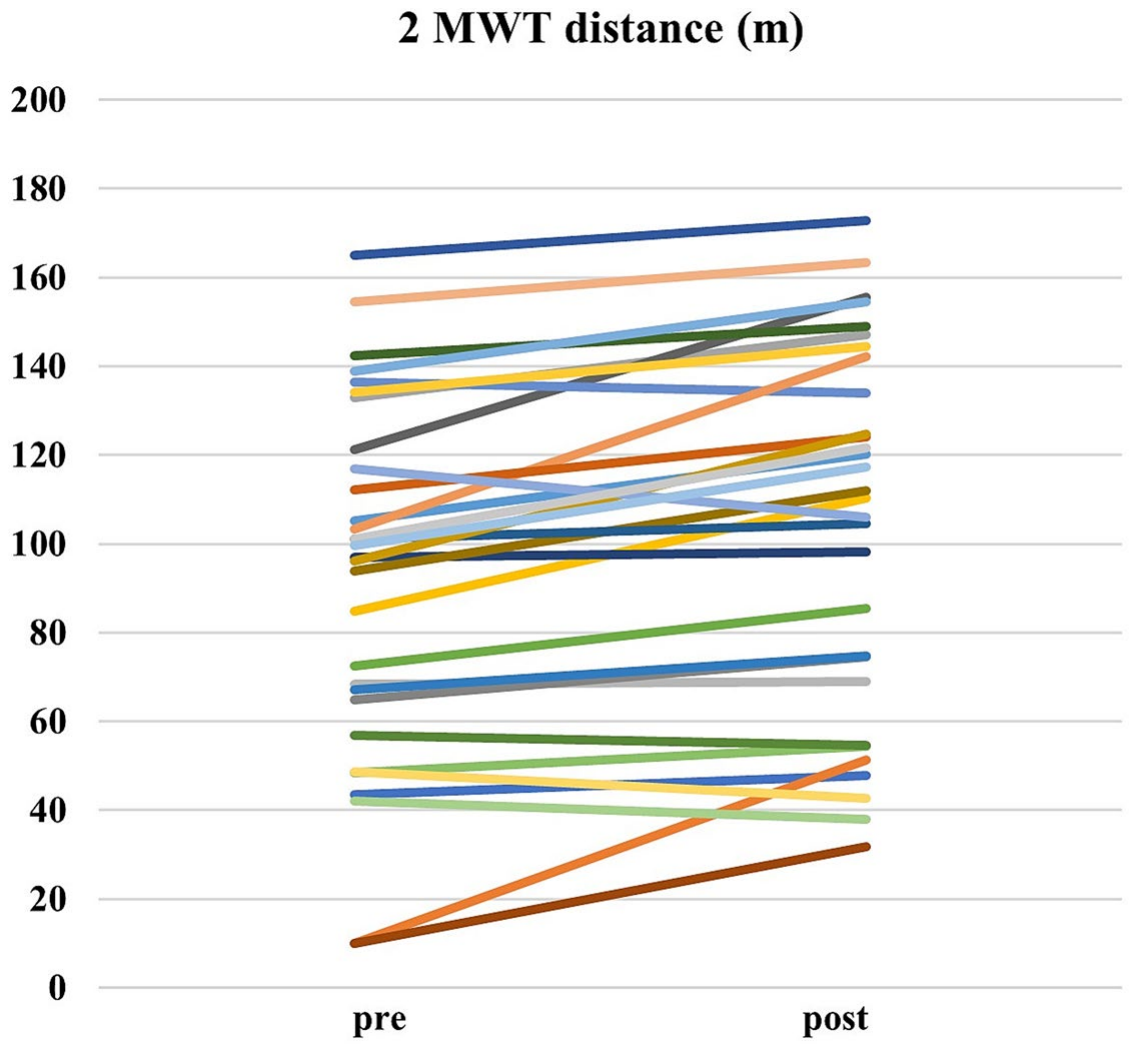
Changes in 2MWT distance before and after HAL treatment in patients with ALS. Each line represents an individual patient, with different shades used to distinguish individuals. The 2MWT distance significantly improved after HAL treatment (*p* < 0.05, paired *t*-test). HAL, Hybrid Assistive Limb; 2MWT, 2-min walk test; ALS, amyotrophic lateral sclerosis.

**Figure 3:**
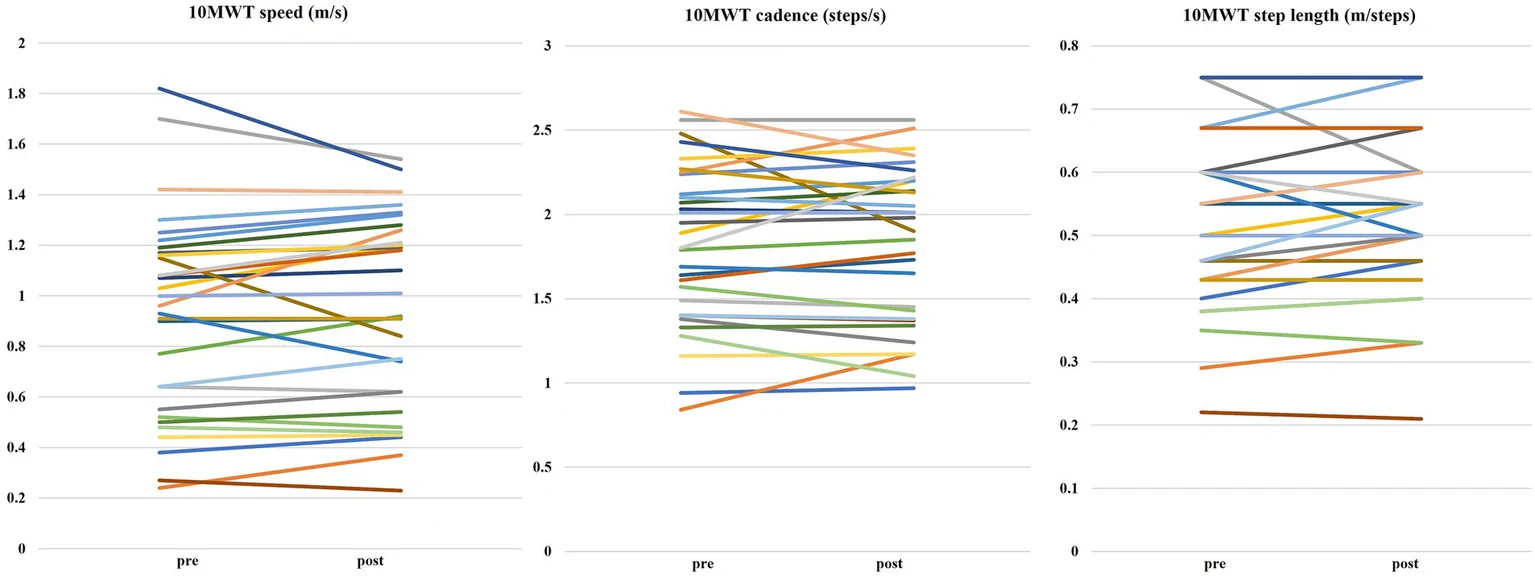
Changes in 10MWT parameters before and after HAL treatment in patients with ALS. Each line represents an individual patient, with different shades used to distinguish individuals. No significant changes were observed in gait speed, cadence, or step length (paired *t*-test). HAL, Hybrid Assistive Limb; 10MWT, 10-m walk test; ALS, amyotrophic lateral sclerosis.

For the SF-36, the physical component summary score (PCS) was 20.82 (10.31), and the role/social component summary score (RCS) was 35.89 (12.81) before HAL treatment, whereas the mental component summary score (MCS) was 55.35 (8.27), indicating relatively preserved mental QoL (Figure 4). Table 3 shows the results before and after treatment. No statistically significant differences were observed in any of the measured outcomes, and the effect sizes were negligible.

**Figure 4:**
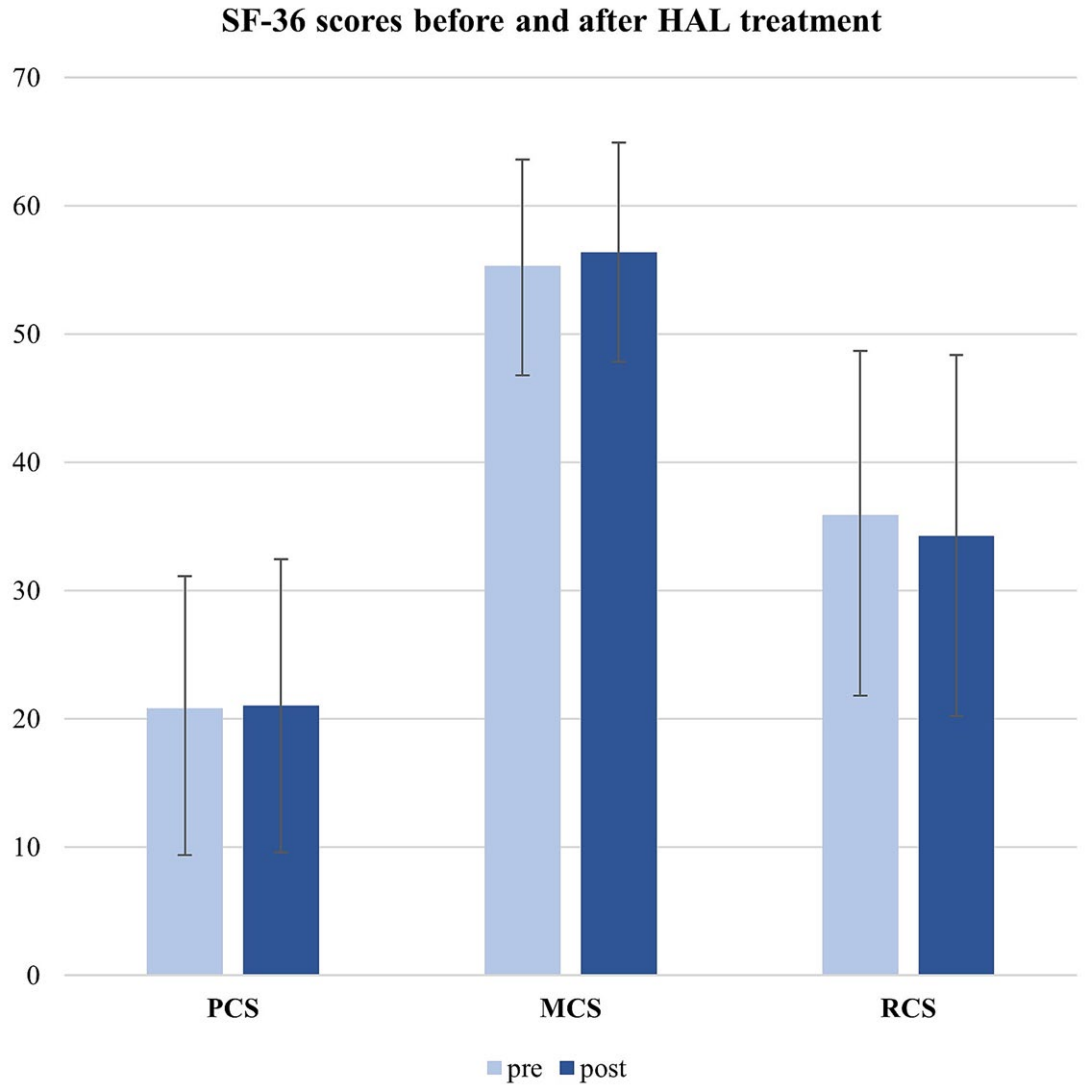
SF-36 scores before and after HAL treatment in patients with ALS. Bar graphs show the mean scores for the PCS, MCS, and RCS. HAL, Hybrid Assistive Limb; SF-36, 36-Item Short Form Health Survey; ALS, amyotrophic lateral sclerosis; PCS, Physical Component Summary; MCS, Mental Component Summary; RCS, Role/Social Component Summary.

**Table 3:** Changes in quality of life scores before and after one course of intervention.

SF-36 component	Baseline	After one course	*p-*value
PCS	20.82	21.02	0.88
MCS	55.35	56.40	0.24
RCS	35.89	34.29	0.39

Data are presented as mean values. Baseline and post-intervention scores were compared using paired *t*-tests. No statistically significant differences were observed. MCS, Mental Component Summary; PCS, Physical Component Summary; RCS, Role/Social Component Summary.

## 4 Discussion

This study investigated the effects of one course of HAL treatment on motor function and QoL in 30 patients with ALS. The results showed a significant improvement in the 2MWT, whereas no significant changes were observed in the 10MWT parameters. Regarding QoL, MCS was relatively preserved and comparable to the national average at baseline, whereas PCS and RCS were lower. No significant changes were observed in any of the SF-36 component scores after HAL treatment.

Several studies from Japan have investigated the effects of HAL treatment on gait function in patients with neuromuscular diseases, including ALS. In the multicenter randomized controlled crossover trial NCY-3001, 30 patients with slowly progressive neuromuscular diseases underwent HAL-assisted gait training or conventional gait training using a hoist. HAL treatment resulted in a significant improvement in the 2MWT compared with the control treatment, providing evidence for the efficacy of HAL in improving ambulatory function in patients with neuromuscular diseases (33). Miura et al. evaluated 10 patients with neuromuscular diseases who underwent two courses of HAL treatment over 6 months and reported significant improvements in the 2MWT as well as in the vitality domain and MCS of the SF-36 (34). In our previous long-term study, three patients with ALS underwent repeated courses of HAL treatment, and gait function was generally maintained during the observation period despite the progressive nature of ALS (35). In another study specifically involving patients with ALS, one course of HAL treatment significantly improved the 2MWT, and cadence also improved significantly, whereas gait speed and step length did not change significantly (36). These findings are generally consistent with the present results, in which the 2MWT improved significantly despite no significant changes in the 10MWT parameters. The present study extends these previous findings by evaluating both gait function and QoL in a larger cohort of patients with ALS.

Evidence regarding the effects of HAL treatment on QoL remains limited. Miura et al. reported that repeated HAL treatment was associated with improvements in the vitality domain and MCS of the SF-36 in patients with neuromuscular diseases (34). In contrast, no significant changes were observed in PCS, MCS, or RCS in the present study. This discrepancy may partly reflect differences in treatment exposure and study populations. Miura et al. evaluated two courses of HAL treatment over 6 months in patients with various neuromuscular diseases, whereas the present study evaluated short-term changes after a single course specifically in patients with ALS. Changes in patient-reported outcomes may require a longer observation period or repeated interventions to become apparent.

In the present study, PCS and RCS were below the national average at baseline, whereas MCS was relatively preserved. Previous studies have shown that QoL in patients with ALS is influenced by multiple factors, including disability, psychological well-being, and patients’ experiences of rehabilitation and daily life (1–3). The relationship between motor function and QoL in patients with ALS is therefore complex, and changes in physical function do not necessarily translate directly into changes in QoL. In particular, psychological well-being may remain relatively preserved despite progressive physical disability (3). Accordingly, the absence of a significant change in QoL in the present study may reflect both the multifactorial nature of QoL and the relatively short duration of the intervention.

The 10MWT and 2MWT were used to assess different aspects of gait performance. The 10MWT primarily reflects short-distance walking speed, whereas the 2MWT evaluates gait performance over a 2-min period and incorporates aspects of both walking speed and endurance. The discrepancy between these outcomes may therefore reflect differences in the aspects of gait being measured rather than inconsistent effects of the intervention. Although the increase of approximately 12 m in the 2MWT reached statistical significance, its clinical relevance remains uncertain. To our knowledge, no established minimal clinically important difference (MCID) for the 2MWT in patients with ALS has been reported. In patients with subacute stroke, the MCID for the 2MWT has been reported to be approximately 31–33 m (38, 39), which is substantially larger than the change observed in the present study. In addition, in patients with neuromuscular diseases, the minimal detectable difference has been reported to be approximately 13.7 m (40), which is close to the magnitude of the change observed in the present study. Therefore, the clinical significance of the observed improvement should be interpreted with caution. Nevertheless, the change may reflect short-term functional responsiveness to HAL treatment. In a progressive disease such as ALS, in which functional decline is expected over time, even small or transient improvements in gait performance may provide useful information regarding residual motor capacity and potential responsiveness to rehabilitation.

From a mechanistic perspective, HAL treatment is based on the concept of cybernics, in which BES generated by voluntary movement are used to drive assisted motion, forming a feedback loop between motor intention and sensory input (41). Repetitive, intention-driven movements using this feedback system may facilitate activity- dependent neural activation and motor learning, consistent with principles of experience-dependent neuroplasticity (42). However, ALS is a progressive neurodegenerative disease, and the extent to which such mechanisms contribute to the observed functional changes remains uncertain. Therefore, the improvement in gait performance observed in the present study should not be interpreted as evidence of disease modification. Rather, HAL may facilitate more efficient use of residual motor function while reducing excessive physical effort during repetitive gait training.

This study has several limitations. First, this was a single-arm observational study without a control group; therefore, the observed changes cannot be conclusively attributed to HAL intervention alone. In particular, practice effects and measurement variability cannot be excluded as explanations for the improvement in the 2MWT. Second, the study period was relatively short, and the long-term impact of HAL treatment on QoL remains unclear. Third, although the present cohort of 30 patients is relatively large compared with previous HAL studies specifically involving patients with ALS, the sample size remains limited. Finally, the present study evaluated a single course of HAL treatment, whereas previous studies suggest that repeated courses may be necessary to detect longer-term changes in gait function or patient-reported outcomes. Further controlled studies with larger sample sizes and longer follow-up periods are required to clarify the effects of HAL treatment on functional outcomes and QoL in patients with ALS.

## 5 Conclusion

In conclusion, HAL treatment was associated with short-term improvements in gait performance in patients with ALS; however, its clinical significance remains uncertain. No significant changes in QoL were observed, highlighting the complex relationship between motor function and patient- reported outcomes in patients with ALS. Further studies are required to determine the long-term effects of HAL treatment on functional outcomes and QoL.

## Data Availability

The original contributions presented in the study are included in the article/supplementary material, further inquiries can be directed to the corresponding author/s.
